# Washed Microbiota Transplantation Lowers Blood Pressure in Patients With Hypertension

**DOI:** 10.3389/fcimb.2021.679624

**Published:** 2021-08-11

**Authors:** Hao-Jie Zhong, Hong-Lie Zeng, Ying-Li Cai, Yu-Pei Zhuang, Yu-Ligh Liou, Qingping Wu, Xing-Xiang He

**Affiliations:** ^1^Department of Gastroenterology, Research Center for Engineering Techniques of Microbiota-Targeted Therapies of Guangdong Province, The First Affiliated Hospital of Guangdong Pharmaceutical University, Guangzhou, China; ^2^Guangdong Provincial Key Laboratory of Microbial Safety and Health, State Key Laboratory of Applied Microbiology Southern China, Institute of Microbiology, Guangdong Academy of Sciences, Guangzhou, China; ^3^School of Biology and Biological Engineering, South China University of Technology, Guangzhou, China; ^4^Xiangya Medical Laboratory, Central South University, Changsha, China

**Keywords:** washed microbiota transplantation, hypertension, treatment, gut microbiota, blood pressure

## Abstract

**Background:**

Although transplantation of the fecal microbiota from normotensive donors has been shown to have an antihypertensive effect in hypertensive animal models, its effect on blood pressure in patients with hypertension is unclear. This study aimed to assess the effect of washed microbiota transplantation (WMT) from normotensive donors on blood pressure regulation in hypertensive patients.

**Methods:**

The clinical data of consecutive patients treated with washed microbiota transplantation (WMT) were collected retrospectively. The blood pressures of hypertensive patients before and after WMT were compared. The factors influencing the antihypertensive effect of WMT in hypertensive patients and fecal microbial composition of donors and hypertensive patients were also analyzed.

**Results:**

WMT exhibited an antihypertensive effect on blood pressure: the blood pressure at hospital discharge was significantly lower than that at hospital admission (change in systolic blood pressure: −5.09 ± 15.51, P = 0.009; change in diastolic blood pressure: −7.74 ± 10.42, P < 0.001). Hypertensive patients who underwent WMT *via* the lower gastrointestinal tract (β = −8.308, standard error = 3.856, P = 0.036) and those not taking antihypertensive drugs (β = −8.969, standard error = 4.256, P = 0.040) had a greater decrease in systolic blood pressure, and hypertensive patients not taking antihypertensive drugs also had a greater decrease in diastolic blood pressure (β = −8.637, standard error = 2.861, P = 0.004). After WMT, the Shannon Diversity Index was higher in six of eight hypertensive patients and the microbial composition of post-WMT samples tended to be closer to that of donor samples.

**Conclusion:**

WMT had a blood pressure-lowering effect in hypertensive patients, especially in those who underwent WMT *via* the lower gastrointestinal tract and in those not taking antihypertensive drugs. Therefore, modulation of the gut microbiota by WMT may offer a novel approach for hypertension treatment.

## Introduction

Hypertension is an independent risk factor for cardiovascular disease, which is the leading cause of death worldwide, and hypertension affects approximately one-third of the world’s adult population (Mills et al., 2020; Valenzuela et al., 2021). The pathogenesis of essential hypertension is complex, involving genetic and lifestyle factors, and the precise mechanisms are incompletely understood (Oparil et al., 2018; Valenzuela et al., 2021). Nevertheless, <5% of the total variance in blood pressure can be explained by common genetic variants (Giri et al., 2019).

Recently, several lines of evidence have suggested a role for the gut microbiota in the regulation of blood pressure. For example, gut dysbiosis (which involves a decreased abundance of probiotic bacteria and increased abundance of pathogenic bacteria) in animal models and in hypertensive patients has been reported (Li et al., 2017; Marques et al., 2018; Yang et al., 2018). In addition, the causative role of the gut microbiota in hypertension pathogenesis has been revealed by fecal microbiota transplantation (FMT) experiments; germ-free mice had significantly increased blood pressure after receiving FMT from hypertensive patients, and Wistar Kyoto (WKY) rats had significantly increased blood pressure after receiving FMT from spontaneously hypertensive rats (SHRs) (Li et al., 2017; Wilck et al., 2017). Moreover, interventions that target the gut microbiota, such as probiotics, antibiotics, and other dietary supplements, have been shown to have antihypertensive effects in animal models (Marques et al., 2017; Wang et al., 2017; Wilck et al., 2017), though the effects were modest in hypertensive patients (Lewis-Mikhael et al., 2020). Furthermore, transfer of fecal samples from normotensive WKY rats to hypertensive SHRs reduced blood pressure significantly in recipients (Toral et al., 2019b). However, whether FMT has a blood pressure-lowering effect in patients is not known.

FMT is an intervention to restore the gut microbiome of patients by transfer of gut-microbiota samples from healthy donors to patients (Bafeta et al., 2017). FMT may be useful for treating several diseases, including *Clostridium difficile* infection, inflammatory bowel disease, and hepatic encephalopathy (Zhang et al., 2020b). However, the high prevalence of adverse events (AEs) and complicated preparation procedure for manually prepared FMT samples limit their use (Ding et al., 2019). A recent study showed that washed microbiota transplantation (WMT), in which fecal particles, parasite eggs, and fungi in the fecal suspension are removed by an automatic machine, reduced FMT-related AEs significantly (Zhang et al., 2020b). Here, we aimed to assess the effect of WMT on blood-pressure regulation in hypertensive patients.

## Materials and Methods

### Study Design and Participants

This retrospective study was undertaken at the First Affiliated Hospital of Guangdong Pharmaceutical University (Guangzhou, China). The study protocol was approved (2021-9) by the Ethics Committee of the First Affiliated Hospital of Guangdong Pharmaceutical University. It was conducted in accordance with the Declaration of Helsinki 1964 and its later amendments. Written informed consent from patients was waived by the Ethics Committee due to the retrospective nature of the study.

Consecutive inpatients who underwent WMT more than once at our hospital from 1 January 2017 to 31 August 2020 were eligible for inclusion. Pregnant patients, patients aged <18 years, and patients who had a change in their regimen of antihypertensive medication after WMT were excluded.

“Hypertension” was defined as office systolic blood pressure (SBP) ≥140 mmHg and/or diastolic blood pressure (DBP) ≥90 mmHg (Williams et al., 2018). Patients without hypertension and shock were considered to be normotensive patients. “Alcoholism” was defined as weekly alcohol intake >140 g in women and >210 g in men. The homeostasis model assessment of insulin resistance (HOMA–IR) was calculated, as described previously (Matthews et al., 1985).

With regard to the indications for WMT, organic diseases (i.e., diseases which led to structural changes in organs or tissues) included inflammatory bowel disease, chronic liver disease, and gastroesophageal reflux disease. Functional diseases (i.e., diseases which did not cause structural changes in organs or tissues) were functional bowel disorder, functional dyspepsia, and gut dysbiosis.

Based on the inclusion and exclusion criteria, 260 patients (73 hypertensive patients and 187 normotensive patients) were included in our study. All of them (n = 260) underwent at least one WMT procedure and completed follow-up, and 72 of them (19 hypertensive patients and 53 normotensive patients) underwent at least two WMT procedures and completed follow-up.

### Preparation and Transplantation of Washed Microbiota

Stools from healthy donors for WMT were screened, as described previously (Zhang et al., 2020a). Briefly, healthy donors were screened with a questionnaire, and then stools and blood were tested to rule out an infectious disease or communicable disease. For preparation of washed microbiota, each 100 g of feces with 500 mL of 0.9% saline was used for making a homogeneous fecal suspension. Then, the fecal suspension was microfiltered (to remove fecal particles, parasite eggs, and fungi) using an automatic machine (GenFMTer; FMT Medical, Nanjing, China). After microfiltration, the fecal supernatant was centrifuged at 1100 × *g* for 3 min at room temperature. Then, the supernatant obtained after centrifugation was discarded. The microbiota pellet was resuspended in saline, followed by centrifugation and resuspension of the microbiota pellet three times. In the final resuspension, 100 mL of saline was added to the microbiota pellet obtained from 100 g of feces (Zhang et al., 2020b). Thereafter, the fecal suspension was infused into patients (120 mL per day for 3 consecutive days) *via* a nasojejunal tube (upper gastrointestinal tract) or a transendoscopic enteral tube (lower gastrointestinal tract) based on the physical status and willingness of each patient (Mullish et al., 2018). Fecal suspensions from the various donors were allocated randomly to patients.

### Data Collection

The following data were extracted from the electronic medical records of patients: age, sex, body mass index (BMI), smoking status, alcoholism, essential hypertension, blood pressure (SBP and DBP), complications of hypertension, use of antihypertensive drugs, indication for WMT, route of WMT delivery (upper or lower gastrointestinal tract), AEs of WMT, and laboratory test results (serum lipids (total cholesterol, triglyceride, high-density lipoprotein [HDL], and low-density lipoprotein [LDL]), fasting glucose, and fasting insulin). Blood pressure upon admission and discharge from hospital was recorded for each WMT procedure.

Blood pressure measured upon hospital admission was defined as “blood pressure at hospital admission”. Blood pressure measured for the last time before hospital discharge was defined as “blood pressure at hospital discharge”. During hospitalization, blood pressure was measured each morning. Because of the fluctuation in blood pressure, the mean blood pressure before WMT (based on ≥2 days of measurements between hospital admission and FMT) and after WMT (based on ≥2 days of measurements between FMT and hospital discharge) were also collected. The antihypertensive effects of WMT were assessed by determining the improvements in blood pressure: △blood pressure = blood pressure at hospital discharge minus blood pressure at hospital admission, and △mean blood pressure = mean blood pressure after the first or second WMT minus mean blood pressure before the first WMT.

### Sample Collection, DNA Extraction and MiSeq Sequencing

Fecal samples were collected for sequencing from 21 donors and eight hypertensive patients before and after WMT. All samples were stored at −80°C after collection until DNA extraction. Microbial DNA was extracted using the E.Z.N.A.^®^ Soil DNA Kit (Omega Biotek, Norcross, GA, USA) according to manufacturer instructions. The quality and concentration of DNA was checked by a spectrophotometer (NanoDrop™ 2000; Thermo Fisher Scientific, Wilmington, DE, USA). Bacterial 16S rRNA gene fragments (V3–V4) were amplified from extracted DNA using primers 338F (5’-ACTCCTACGGGAGGCAGCAG-3’) and 806R (5’-GGACTACHVGGGTWTCTAAT-3’) and the following polymerase chain reaction (PCR conditions): 30 s at 95°C, 30 s at 55°C, and 45 s at 72°C for 25 cycles. PCRs were carried out with 4 μL of 5× *TransStart* FastPfu buffer, 2 μL of 2.5 mM deoxynucleoside triphosphates, 0.8 μL of each primer (5 μM), 0.4 μL *TransStart* FastPfu DNA Polymerase, 10 ng of extracted DNA and, finally, with double-distilled water to make the volume up 20 μL. Agarose gel electrophoresis was undertaken to ascertain the size of amplicons. The latter were subjected to paired-end sequencing on the MiSeq sequencing platform (Illumina, San Diego, CA, USA) using PE300 chemical at Majorbio BioPharm Technology (Shanghai, China).

### Sequence Processing and Analyses of Amplicons

After demultiplexing, the resulting sequences were merged with FLASH (v1.2.11) (Magoc and Salzberg, 2011) and quality filtered with fastp (0.19.6) (Chen et al., 2018). Then, high-quality sequences were de-noised using the DADA2 plugin (Callahan et al., 2016) in the Qiime2 (version 2020.2) (Bolyen et al., 2019) pipeline using recommended parameters. These actions led to obtaining single nucleotide resolution based on error profiles within samples. Usually, DADA2-denoised sequences are called “amplicon sequence variants” (ASVs). Taxonomic assignment of ASVs was carried out using the Naive Bayes consensus taxonomy classifier implemented in Qiime2 and the SILVA 16S rRNA database (v138). Analyses of 16S rRNA microbiome sequencing data were undertaken using the free online platform of Majorbio Cloud Platform (www.majorbio.com/).

### Statistical Analyses

Statistical analyses were carried out using SPSS 22.0 (IBM, Armonk, NY, USA) and Prism 8 (GraphPad, San Diego, CA, USA). Results are expressed as frequencies and percentages for categorical variables, mean and SD for continuous variables with a normal distribution, and medians and interquartile ranges for continuous variables with a non-normal distribution. Categorical variables were analyzed using the chi-square test or Fisher’s exact test. For comparisons of continuous variables between two independent groups, the unpaired Student’s *t*-test (for variables with a normal distribution) or the Mann–Whitney *U*-test (for variables with a non-normal distribution) were used. For comparisons of paired data, the paired Student’s *t*-test (for variables with a normal distribution) or the Mann–Whitney *U*-test (for variables with a non-normal distribution) were used. The Wilcoxon rank sum test was employed to detect genera with significant differential abundance in hypertensive patients before and after WMT. To identify the factors (including complications of hypertension, use of antihypertensive drugs, indication for WMT, and delivery route of WMT) that affect the antihypertensive effect of WMT after adjustment for potential confounders, stepwise multivariate linear regression analyses of △SBP and △DBP were conducted using the following independent variables: age, sex, BMI, smoking status, alcoholism, HDL, LDL, complications of hypertension, use of antihypertensive drugs, indication for WMT, and delivery route of WMT. P < 0.05 (two-tailed) was considered significant.

## Results

### Clinical Characteristics of Healthy-Stool Donors and Patients Who Underwent WMT

Healthy-stool donors comprised 25 people (11 males and 14 females) who passed the screening stage. Their median age was 25.0 (interquartile range, 23.0–26.5) years and their mean BMI was 20.1 ± 2.1 kg/m^2^.

A functional gastrointestinal disorder (including functional bowel disorder, functional dyspepsia, and gut dysbiosis) was the most common indication for WMT, accounting for 60.38% (n = 157) of cases, followed by inflammatory bowel disease (n = 30), chronic liver disease (n = 26), gastroesophageal reflux disease (n = 15), metabolic disease (n = 10), neurological and psychiatric disorders (n = 6), radiation-induced enteritis (n = 5), and other disorders (n = 11).

The duration of hospital stay for the first WMT was 8.00 (5.00–10.00) days and the interval between the first WMT and second WMT was 32.00 (26.00–47.50) days. A comparison of the demographics and clinical characteristics between hypertensive patients and normotensive patients is shown in **Table 1**. Among hypertensive patients, 71.23% (52/73) were taking antihypertensive drugs.

**Table 1:** Demographics and clinical characteristics of patients.

	Normotensive patients (N = 187)	Hypertensive patients (N = 73)	P
**Age (years)**	49.00 (38.00–63.00)	65.00 (59.00–74.00)	**<0.001**
**Male sex**	96 (50.79)	38 (52.05)	0.855
**BMI (kg/m^2^)**	21.36 (19.05–23.87) (n = 184)	25.07 (21.42–27.96) (n = 69)	**<0.001**
**Smoking status**			0.114
** Never**	161 (86.10)	55 (75.34)	
** Former**	11 (5.88)	8 (10.96)	
** Current**	15 (8.02)	10 (13.70)	
**Alcoholism**	7 (3.74)	9 (12.33)	**0.021**
**SBP at hospital admission (mmHg)**	120.00 (112.00–127.25) (n = 186)	126.00 (122.00–138.00) (n = 73)	**<0.001**
**DBP at hospital admission (mmHg)**	75.00 (70.00–81.00) (n = 186)	78.00 (75.00–85.00) (n = 73)	**<0.001**
**Total cholesterol (mmol/L)**	4.60 (3.84–5.50) (n = 152)	4.51 (4.04–5.21) (n = 64)	0.506
**Triglyceride (mmol/L)**	0.98 (0.70–1.34) (n = 152)	1.26 (1.00–1.95) (n = 64)	**<0.001**
**LDL (mmol/L)**	2.70 (2.03–3.54) (n = 152)	2.54 (2.04–3.28) (n = 64)	0.320
**HDL (mmol/L)**	1.30 ± 0.3 (n=152)	1.22 ± 0.32 (n=64)	0.139
**HOMA–IR**	1.46 (1.02–2.06) (n = 101)	2.52 (1.46–3.38) (n = 51)	**0.001**

Data are the mean ± standard deviation, median (interquartile range), or n (%). BMI, body mass index; DBP, diastolic blood pressure; HDL, high-density lipoprotein; HOMA–IR, homeostasis model assessment of insulin resistance; LDL, low-density lipoprotein; SBP, systolic blood pressure. Significant P-values are emboldened.

### Effect of WMT on Blood Pressure in Hypertensive Patients and Normotensive Patients

**Figure 1:**
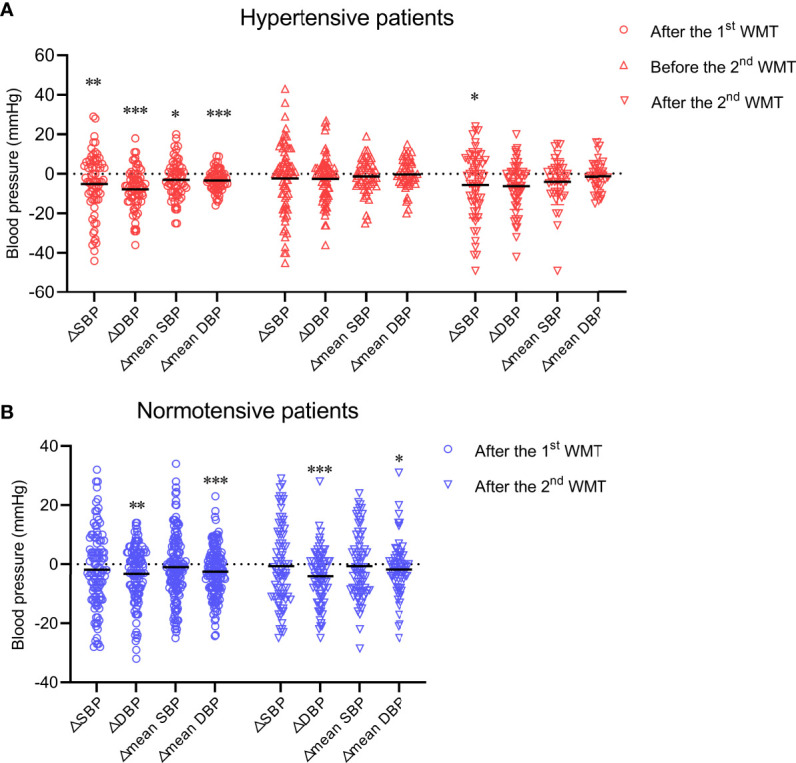
Effect of WMT on blood pressure in hypertensive patients **(A)** and normotensive patients **(B)**. Data are the mean. △SBP: systolic blood pressure at hospital discharge after the first or second WMT or at hospital admission before the second WMT minus systolic blood pressure at hospital admission before the first WMT; △DBP: diastolic blood pressure at hospital discharge after the first or second WMT or at hospital admission before the second WMT minus diastolic blood pressure at hospital admission before the first WMT; △mean SBP: mean systolic blood pressure after the first or second WMT minus mean systolic blood pressure before the first WMT; △mean DBP: mean diastolic blood pressure after the first or second WMT minus mean diastolic blood pressure before the first WMT. WMT, washed microbiota transplantation. *P<0.05, **P<0.01, ***P<0.001.

First, we sought to determine if WMT affects blood pressure in hypertensive cases and normotensive patients. In hypertensive patients, WMT showed a short-term blood pressure-lowering effect. That is, SBP and DBP at hospital discharge after the first WMT were significantly lower than those at hospital admission before the first WMT (△SBP: −5.09 ± 15.51, P = 0.009; △DBP: −7.74 ± 10.42, P < 0.001). The mean SBP and mean DBP after the first WMT were significantly lower than those before the first WMT (△mean SBP: −2.92 ± 9.44, P = 0.015; △mean DBP: −3.27 ± 5.33, P < 0.001) (**Figure 1A**). However, a medium-term blood pressure-lowering effect of WMT was not found. There was no significant difference in blood pressure 1 month after the first WMT (before the second WMT) compared with that before the first WMT (△SBP: −2.23 ± 17.54, P = 0.280; △DBP: −1.00 (−10.00 to 3.00), P = 0.052; △mean SBP: −1.26 ± 8.75, P = 0.318; △mean DBP: −0.22 ± 7.50, P = 0.838) (**Figure 1A**). With regard to the second WMT, there was a significant decrease in SBP at hospital discharge after the second WMT compared with that at hospital admission before the first WMT (△SBP: −5.55 ± 16.73, P = 0.014). DBP at hospital discharge after the second WMT (△DBP: −5.50 (−12.00 to 1.00), P = 0.069) and mean SBP and mean DBP after the second WMT (△mean SBP: −3.00 (−9.75 to 3.00), P = 0.051; △mean DBP: −1.31 ± 7.83, P = 0.298) were lower than the corresponding values before the first WMT, although the differences were not significant (**Figure 1A**).

In normotensive patients, although WMT led to a modest blood pressure-lowering effect with regard to △DBP (first WMT: –3.21 ± 9.62, P = 0.001; second WMT: −2.51 ± 8.29, P < 0.001) and △mean DBP (first WMT: −2.51 ± 8.29, P < 0.001; second WMT: −2.00 (−5.40 to 2.00), P = 0.011), SBP was not affected significantly by WMT (**Figure 1B**).

### Effect of WMT on Blood Pressure in Hypertensive Patients With Different Characteristics

**Figure 2:**
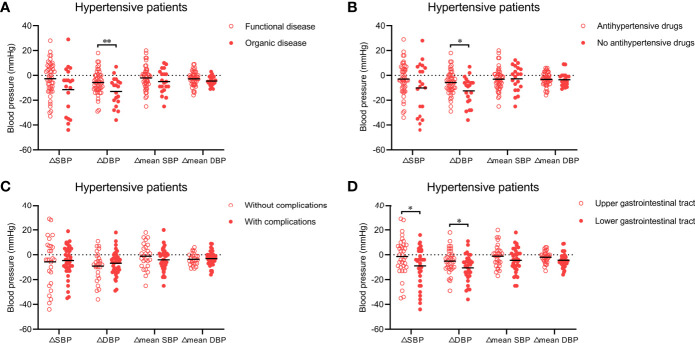
**(A)** Effect of WMT on blood pressure in hypertensive patients who underwent WMT to treat organic disease or functional disease. **(B)** Effect of WMT on blood pressure in hypertensive patients taking or not taking antihypertensive drugs. **(C)** Effect of WMT on blood pressure in hypertensive patients with or without complications. **(D)** Effect of the delivery route of WMT on blood pressure in hypertensive patients. Data are the mean. △SBP: systolic blood pressure at hospital discharge minus systolic blood pressure at hospital admission; △DBP: diastolic blood pressure at hospital discharge minus diastolic blood pressure at hospital admission; △mean SBP: mean systolic blood pressure after the first WMT minus mean systolic blood pressure before the first WMT; △mean DBP: mean diastolic blood pressure after the first WMT minus mean diastolic blood pressure before the first WMT. WMT, washed microbiota transplantation. *P<0.05, **P<0.01.

Next, we analyzed which types of hypertensive patients (n = 73) had a more significant improvement in blood pressure (△SBP, △DBP, △mean SBP, and △mean DBP) after WMT. Twenty patients and 53 hypertensive patients underwent WMT to treat an organic disease (e.g., inflammatory bowel disease, chronic liver disease, and gastroesophageal reflux disease) and functional diseases (e.g., functional bowel disorder, functional dyspepsia, and gut dysbiosis), respectively. **Figure 2A** shows that WMT seemed to have a better antihypertensive effect in hypertensive patients who underwent WMT to treat organic diseases than in those who underwent WMT to treat functional diseases (△SBP: −11.42 ± 19.30 *vs*. −2.63 ± 13.20, P = 0.080; △DBP: −13.00 ± 11.46 *vs*. −5.69 ± 9.33, P = 0.008), although there was no significant difference in △mean SBP (−4.94 ± 9.53 *vs*. −2.02 ± 9.37, P = 0.253) or △mean DBP (−4.57 ± 3.80 *vs*. −2.69 ± 5.82, P = 0.192) between the two groups.

A total of 52 hypertensive patients were taking antihypertensive drugs and 21 were not. Patients who were not taking antihypertensive drugs had higher △SBP (−10.10 ± 20.33 *vs*. −3.00 ± 12.68, P = 0.160) and △DBP (−12.50 ± 11.49 *vs*. −5.75 ± 9.36, P = 0.014) than those taking antihypertensive drugs, but there was no significant difference in △mean SBP (−2.69 ± 10.30 *vs*. −3.02 ± 9.16, P = 0.896) or △mean DBP (−3.47 ± 5.54 *vs*. −3.18 ± 5.29, P = 0.838) between the two groups (**Figure 2B**).

With regard to the complications of hypertension, 44 hypertensive patients had at least one complication (e.g., stroke, coronary heart disease, or heart failure) and 29 had none. △SBP, △DBP, △mean SBP, and △mean DBP were not significantly different between hypertensive patients with or without complications (**Figure 2C**).

### Effect of the Delivery Route of WMT on Blood Pressure in Hypertensive Patients

We also explored whether the antihypertensive effect of WMT was affected by the delivery route. Among hypertensive patients, 37 received WMT *via* the upper gastrointestinal tract and 36 received WMT *via* the lower gastrointestinal tract. Compared with the former, the latter led to a significantly higher △SBP (−9.00 ± 15.28 *vs*. −1.40 ± 15.02, P = 0.043) and △DBP (−10.55 ± 10.74 *vs*. −5.09 ± 9.51, P = 0.030), and non-significantly higher △mean SBP (−4.46 ± 9.70 *vs*. −1.12 ± 8.96, P = 0.157) and △mean DBP (−4.41 ± 5.67 *vs*. −1.93 ± 4.73, P = 0.061) (**Figure 2D**).

The multivariate linear regression analysis, which adjusted for potential confounders, showed that hypertensive patients who underwent WMT *via* the lower gastrointestinal tract (β = −8.308, standard error [SE] = 3.856, P = 0.036) and hypertensive patients who underwent WMT and did not take antihypertensive drugs (β = −8.969, SE = 4.256, P = 0.040) had higher △SBP. Hypertensive patients who underwent WMT and did not take antihypertensive drugs (β = −8.637, SE = 2.861, P = 0.004) also had higher △DBP. However, other factors had no influence on the change in blood pressure with WMT treatment.

### Effect of WMT on Hypertension-Related Risk Factors in Hypertensive Patients

Furthermore, the effects of WMT on hypertension-related risk factors (i.e., BMI, HOMA–IR, blood lipids) were assessed. **Table 2** shows that WMT did not alter any of these variables significantly.

**Table 2:** Effect of WMT on hypertension-related risk factors in hypertensive patients.

	Before the first WMT	After the first WMT	P	Before the first WMT	After the second WMT	P
**BMI (kg/m^2^)**	24.71 ± 3.96 (n = 68)	24.43 ± 4.23 (n = 68)	0.284	25.34 ± 3.69 (n = 37)	25.25 ± 3.98 (n = 37)	0.821
**HOMA–IR**	2.38 (1.48–3.18) (n = 26)	2.72 (1.44–4.97) (n = 26)	0.112	2.76 (1.53–3.68) (n = 20)	2.61 (1.28–4.79) (n = 20)	0.852
**Total cholesterol (mmol/L)**	4.70 ± 0.93 (n = 44)	4.75 ± 0.96 (n = 44)	0.716	4.54 (3.92–5.64) (n = 28)	4.22 (3.81–4.78) (n = 28)	0.175
**Triglyceride (mmol/L)**	1.37 (1.08–2.14) (n = 44)	1.50 (0.93–2.09) (n = 44)	0.809	1.38 (1.14–2.51) (n = 28)	1.31 (0.90–2.38) (n = 28)	0.113
**HDL (mmol/L)**	1.18 (1.02–1.38) (n = 44)	1.21 (1.07–1.39) (n = 44)	0.890	1.19 (0.98–1.35) (n = 28)	1.15 (1.01–1.32) (n = 28)	0.269
**LDL (mmol/L)**	2.62 (2.07–3.33) (n = 44)	2.79 (2.15–3.43) (n = 44)	0.432	2.46 (2.07–3.34) (n = 28)	2.34 (1.96–2.94) (n = 28)	0.050

Data are the mean ± standard deviation or median (interquartile range). BMI, body mass index; WMT, washed microbiota transplantation; HDL, high-density lipoprotein; HOMA–IR, homeostasis model assessment of insulin resistance; LDL, low-density lipoprotein.

### AE Prevalence in Patients Who Underwent WMT

We also analyzed AE prevalence in patients who underwent WMT. WMT-related AEs were identified based on clinical judgment and all available information (mainly fever, diarrhea, nausea, vomiting, abdominal pain, bloating, dizziness, headache, and anal pain). A total of 520 WMT procedures were analyzed, and the overall prevalence of AEs was 3.07%. Abdominal pain and bloating were the most common AEs (six WMT procedures, 1.15%), followed by diarrhea (five WMT procedures, 0.96%), dizziness (three WMT procedures, 0.58%), nausea (one WMT procedure, 0.19%), and anal pain (one WMT procedure, 0.19%).

### Analyses of Microbiota Composition

**Figure 3:**
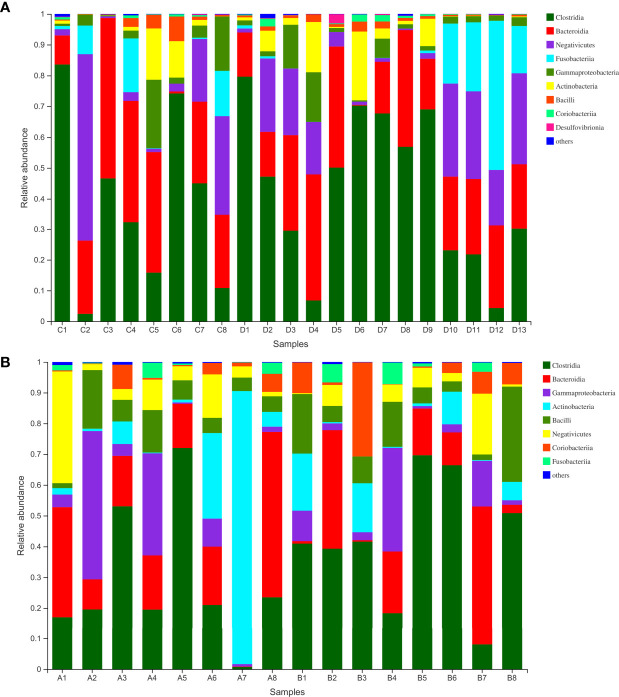
**(A)** Class-level abundance in WMT donors. **(B)** Class-level abundance in hypertensive patients before and after WMT (sample A1–8: before WMT, B1–8: after WMT). WMT, washed microbiota transplantation.

**Figure 4:**
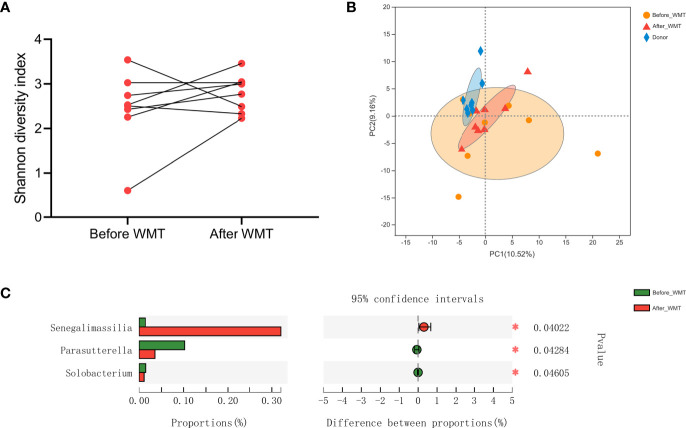
**(A)** Shannon Diversity Index in hypertensive patients before and after WMT. **(B)** Principal component analysis of microbial taxa abundance from WMT donors and hypertensive patients before and after WMT. **(C)** Relative abundance of genus significantly different between hypertensive patients before and after WMT. WMT, washed microbiota transplantation.

Finally, we analyzed the microbiota composition of fecal samples from 21 WMT donors and eight hypertensive patients before and after WMT. Their taxonomic profiles are shown in **Figure 3**. After WMT, the Shannon index was increased in six of eight hypertensive patients (**Figure 4A**). Principal component analysis of taxonomic abundance showed that post-WMT samples tended to be closer to donor samples, although this did not reach significance, probably due to the small sample size (analysis of similarities: before WMT *vs*. after WMT, R = 0.031, P = 0.273; before WMT *vs*. donors, R = −0.002, P = 0.476; after WMT *vs*. donors, R = 0.054, P = 0.159) (**Figure 4B**). Compared with baseline, patients after WMT had marked changes in genus-level relative abundance, including an increased abundance of *Senegalimassilia* species, and decreased abundance of *Parasutterella* and *Solobacterium* species (**Figure 4C**).

## Discussion

WMT had a blood pressure-lowering effect in hypertensive patients (especially in those who underwent WMT *via* the lower gastrointestinal tract and those who did not use antihypertensive drugs) but WMT had virtually no effect on the blood pressure of normotensive patients. This is the first clinical study to determine the antihypertensive effect of WMT in hypertensive patients. Our data suggest that manipulation of the gut microbiota could be a new therapeutic avenue for hypertension.

Several intervention studies have shown that blood pressure in hypertensive animal models can be modified by altering the gut microbiota (e.g., using antibiotics, prebiotics, or probiotics) (Yang et al., 2015; Marques et al., 2017; Wang et al., 2017). For example, Yang and colleagues showed that oral administration of the antibiotic minocycline for 4 weeks in a hypertensive animal model restored and reshaped the microbiota composition and lowered blood pressure significantly (Yang et al., 2015). In addition, high fiber and acetate supplementation have been reported to modify the gut microbiota, increase the abundance of acetate-producing bacteria, and prevent the development of hypertension in deoxycorticosterone acetate–salt-induced hypertensive mice (Marques et al., 2017). Although several animal and clinical studies have been conducted to evaluate the effect of probiotics on hypertension (Wilck et al., 2017; Lewis-Mikhael et al., 2020; Verhaar et al., 2020), a recent meta-analysis of seven randomized controlled trials involving 653 participants concluded that supplementation with *Lactobacillus plantarum* resulted in a significant but modest reduction in DBP (−0.92 mmHg; 95%CI: −1.49 to −0.35) and no significant reduction in SBP (−1.58 mmHg; 95%CI: −3.05 to 0.11), indicating that the blood pressure-lowering effect of probiotic supplementation might not be clinically significant (Lewis-Mikhael et al., 2020). In addition, another meta-analysis on the antihypertensive effect of probiotics revealed that multispecies probiotics had a greater impact on blood-pressure improvement than single-species probiotics (Khalesi et al., 2014). As a type of multispecies gut-microbiota transplantation, FMT has been reported to have markedly antihypertensive effects in hypertensive animals (Toral et al., 2019b). Consistently, our results showed a similar effect in hypertensive patients. However, our study showed that WMT had a short-term (3–7 days) but not medium-term (1 month) blood pressure-lowering effect. Although the antihypertensive effect of WMT was temporary, it was longer than the effects of conventional antihypertensive medications. Further studies are needed to explore how to prolong the antihypertensive effect of WMT.

Several clinical studies have found that the gut microbiota in hypertensive patients differs significantly from that in healthy controls, and is characterized by loss of microbial diversity, loss of beneficial bacteria, and expansion of potentially harmful bacterial (Li et al., 2017; Yan et al., 2017). Our data for 16S rRNA sequencing showed that WMT might restore the microbial diversity in hypertensive patients and modulate their microbial composition similar to that observed in healthy controls. Samples from only eight hypertensive patients were obtained for sequencing, so this hypothesis requires further exploration. Besides, we found a significantly higher abundance of the genus *Senegalimassilia* in hypertensive patients after WMT. Adamberg and colleagues showed that a higher abundance of *Senegalimassilia* was associated with healthy traits. They revealed that children without obesity had a higher abundance of *Senegalimassilia anaerobia* compared with those who were overweight (Adamberg et al., 2018). Also, diabetic rats treated with a formula that could ameliorate hyperglycemia significantly also have been shown to have a high abundance of *Senegalimassilia* species (Gao et al., 2018). Furthermore, the abundance of *Parasutterella* species (increased in hypertensive patients) (Mushtaq et al., 2019) and *Solobacterium* species (associated with atherosclerotic cardiovascular disease) (Tierney et al., 2021) was decreased markedly after WMT. Therefore, WMT might lower the blood pressure in hypertensive patients by restoring the balance of the gut microbiota.

Although the other mechanisms involved in the antihypertensive effects of WMT are not clear, four concepts might explain (at least in part) our results. First, a microbiota transplant might influence the host’s physiology through the production of bacterial metabolites. Studies have demonstrated that there is a reduction in circulating short-chain fatty acids (SCFAs) in SHRs and hypertensive patients (Kim et al., 2018; Yang et al., 2019b). FMT from normotensive to hypertensive animals restored the abundance of butyrate-producing bacteria and lowered blood pressure, indicating that FMT might affect the production of gut microbiota-derived SCFAs (which have been shown to play a key part in blood-pressure regulation) (Toral et al., 2019b). Second, the gut microbiota might affect regulation of the circulatory system *via* the enteric nervous system; Toral and coworkers showed that FMT from normotensive rats to SHRs decreased blood pressure and activity of the sympathetic nervous system by reducing neuroinflammation (Toral et al., 2019b). Third, the higher gut permeability of hypertensive rats was lowered by FMT from normotensive rats, indicating that FMT improves gut permeability, reduces endotoxin absorption and, subsequently, modulates blood pressure (Toral et al., 2019b). Finally, a microbiota transplant also lowered blood pressure by restoring the balance of T-helper type 17/T regulatory cells and reducing the production of pro-inflammatory cytokines (Toral et al., 2019a).

The microbes in feces mostly consist of the microbiota in the large intestine. Li and collaborators found that the large-intestinal microbiota was more likely to colonize the large intestine and rarely colonized the small intestine after transplantation (Li et al., 2020b). Their data suggested that the microbiota from one specific location selectively colonizes its homologous intestinal area. In our study, patients who received WMT *via* the lower gastrointestinal tract had taken laxatives to clear intestinal feces, which might also have facilitated colonization of the large intestine by the transplanted microbiota. Accordingly, our study revealed that transplantation of the large-intestinal microbiota into the large intestine of hypertensive patients *via* the lower gastrointestinal tract led to a more obvious blood pressure-lowering effect than that elicited by transplantation into the small intestine *via* the upper gastrointestinal tract. Similarly, Xue and colleagues found that colonic FMT improved Parkinson’s disease significantly and had longer-term effects compared with nasointestinal FMT (Xue et al., 2020).

We found that hypertensive patients who were not taking antihypertensive drugs experienced a more pronounced antihypertensive effect after WMT compared with those taking antihypertensive drugs. After taking antihypertensive drugs, the blood pressure of the hypertensive patients could drop to a stable level, so the antihypertensive effect of WMT might have been less obvious. Moreover, some antihypertensive drugs, such as captopril and losartan, have been shown to reduce gut dysbiosis in SHRs (Yang et al., 2019a; Li et al., 2020a; Robles-Vera et al., 2020). Therefore, patients taking antihypertensive drugs might undergo correction of their gut microbiota, and the antihypertensive effect of WMT might be weakened in such patients.

Hypertensive patients with complications often have worse vascular elasticity, which might lead to a poor antihypertensive effect of WMT. However, a significant difference in blood-pressure improvement after WMT was not found between hypertensive patients with or without complications. Moreover, we did not observe significant improvements in hypertension-related risk factors (including HOMA–IR) after WMT, although several studies have shown that FMT improves insulin sensitivity significantly (Vrieze et al., 2012; Kootte et al., 2017). Our non-significant results might have been due to the small sample size in our study and the lack of insulin resistance in many of our hypertensive patients.

The safety of WMT for treating hypertension is crucial. We showed that the prevalence of AEs after WMT was low (3.07%). The main AEs were abdominal pain, bloating, diarrhea, and dizziness. Serious AEs were not observed in the present study: WMT may be safe and efficacious treatment for hypertension.

Our study had four main limitations. First, given that this was a retrospective study, few fecal samples were collected from hypertensive patients, and the fecal metabolism in patients before and after WMT were not assessed. Hence, the mechanism underlying the antihypertensive effect of WMT was not elucidated. Second, this was a single-center study with a small sample size, so the statistical power to detect the effects of WMT on hypertension-related risk factors might have been insufficient. Third, potential confounders which might affect blood pressure in the short term (e.g., sleep quality, psychological state) were not taken into consideration. Fourth, the suspension of washed microbiota was not prepared under anaerobic conditions. Given that most of the beneficial bacteria are strictly anaerobic, some of them might have died during this aerobic preparation, which introduced a bias favoring the growth of aerobic bacteria. Therefore, our findings on the antihypertensive effect of WMT should be interpreted cautiously, and large-scale prospective studies are needed to verify our conclusions.

## Conclusions

WMT had a blood pressure-lowering effect in hypertensive patients, especially in patients who underwent WMT *via* the lower gastrointestinal tract and patients who did not take antihypertensive drugs. Therefore, modulation of the gut microbiota by WMT may offer an interesting and novel approach for hypertension treatment.

## Data Availability Statement

The original contributions presented in the study are publicly available. This data can be found here: NCBI Sequence Read Archive (Accession: PRJNA748720).

## Ethics Statement

The studies involving human participants were reviewed and approved by the Ethics Committee of the First Affiliated Hospital of Guangdong Pharmaceutical University. Written informed consent for participation was not required for this study in accordance with the national legislation and the institutional requirements.

## Author Contributions

QW and X-XH designed the study. H-JZ, H-LZ, Y-LC, and Y-PZ collected and analyzed the data. All authors helped to write the manuscript. All authors contributed to the article and approved the submitted version.

## Funding

This study was supported by the Natural Science Foundation of Guangdong Province (2019A1515010125) and Department of Education of Guangdong Province (2019-GDXK-0013 and 2020KZDZX1132).

## Conflict of Interest

The authors declare that the research was conducted in the absence of any commercial or financial relationships that could be construed as a potential conflict of interest.

## Publisher’s Note

All claims expressed in this article are solely those of the authors and do not necessarily represent those of their affiliated organizations, or those of the publisher, the editors and the reviewers. Any product that may be evaluated in this article, or claim that may be made by its manufacturer, is not guaranteed or endorsed by the publisher.
